# Muscle amnesia: a hypothesized framework for motor-pattern accessibility in exercise rehabilitation after inactivity, injury and illness

**DOI:** 10.3389/fspor.2026.1926470

**Published:** 2026-09-30

**Authors:** Giorgos K. Sakkas, Christina Karatzaferi

**Affiliations:** 1 CREHP, Department of Physical Education and Sport Science, School of Physical Education, Sport Science and Dietetics, University of Thessaly, Trikala, Greece; 2 Department of Life Sciences, School of Life and Health Sciences, University of Nicosia, Nicosia, Cyprus

**Keywords:** detraining, disuse, exercise therapy, motor control, motor learning, movement quality, rehabilitation, return to activity

## Abstract

After inactivity, injury or illness, people may regain part of their strength, range of motion or load tolerance before they recover fluent movement. Clinicians may observe an athlete, patient or older adult who can generate force but returns to walking, squatting, standing, running or sport with a guarded, variable or inefficient movement strategy that appears disproportionate to remaining impairment. This Perspective presents a theoretical framework and proposes the term Muscle Amnesia as a cautious practical label for a history-, use- and context-sensitive reduction in access to a previously acquired task solution. The term is not intended to imply literal memory loss within muscle tissue, to replace established concepts or to create a new diagnosis. Rather, it names a candidate clinical-behavioural construct in which altered exposure, compensatory practice, pain, threat, fatigue, context change and reduced task practice may shift motor-pattern selection. The aim of this article is to define the construct, distinguish it from detraining, learned non-use, pain-adapted movement, skill decay and maladaptive compensation, and propose preliminary candidate criteria and research designs. We argue that exercise rehabilitation should examine not only physical capacity, but also whether capacity is organized into task-relevant movement. The framework remains hypothetical and requires qualitative, experimental and clinical validation.

## Introduction

Exercise rehabilitation often assumes that restored capacity will translate into restored function. In many cases, this assumption is correct: progressive loading, aerobic conditioning, nutrition, pain control and graded exposure improve strength, endurance, confidence and activity tolerance. However, clinical exercise practitioners, physiotherapists and coaches also encounter a residual mismatch. After inactivity, injury or illness, an individual may recover measurable capacity yet still execute a familiar task with excessive guarding, poor timing, instability, increased variability or an inefficient compensatory movement strategy. The individual may describe the experience informally as: “I know I used to do this, but my body no longer finds the movement.” This phrase is used here as an illustrative clinical observation, not as empirical evidence.

Existing constructs capture important parts of this problem. Detraining describes the loss of training-induced physiological and performance adaptations after an insufficient training stimulus (1, 2). Dynapenia and sarcopenia emphasize that strength and physical performance can decline beyond what is explained by muscle size alone (3, 4). Learned non-use, particularly in neurorehabilitation, describes behaviour that persists because affected movement is avoided or insufficiently reinforced (5). Pain-adapted movement theory explains why movement may reorganize under pain or threat and why such adaptation can remain clinically relevant (6). Return-to-sport consensus statements also increasingly emphasize that strength, range of motion, neuromuscular control, psychological readiness and movement quality must be considered together (7).

Despite these related ideas, none specifically names a post-exposure state in which a previously successful movement strategy becomes difficult to access while the person's capacity is partly preserved or recovering. We propose Muscle Amnesia as a memorable but deliberately cautious term for this state. The novelty of the term is not a claim that the underlying mechanisms are new. Its potential value is translational: it draws attention to motor-pattern accessibility as a rehabilitation target that is distinct from, but interacts with, strength, range of motion, pain, tissue healing, fatigue and confidence.

## Core terminology and scope

We define Muscle Amnesia as a hypothesized clinical-behavioural construct: a history-dependent, use- and context-sensitive reduction in access to a previously acquired task solution, not fully explained by loss of force-generating capacity, range of motion or tissue integrity, and only provisionally supported by improvement after task-specific re-exposure (1–4, 6). The construct is not a diagnosis and should not be used to medicalize normal movement variability. It is a research hypothesis intended to help clinicians and investigators ask whether a person can still access a task solution that previously supported functional movement (Figure 1).

**Figure 1:**
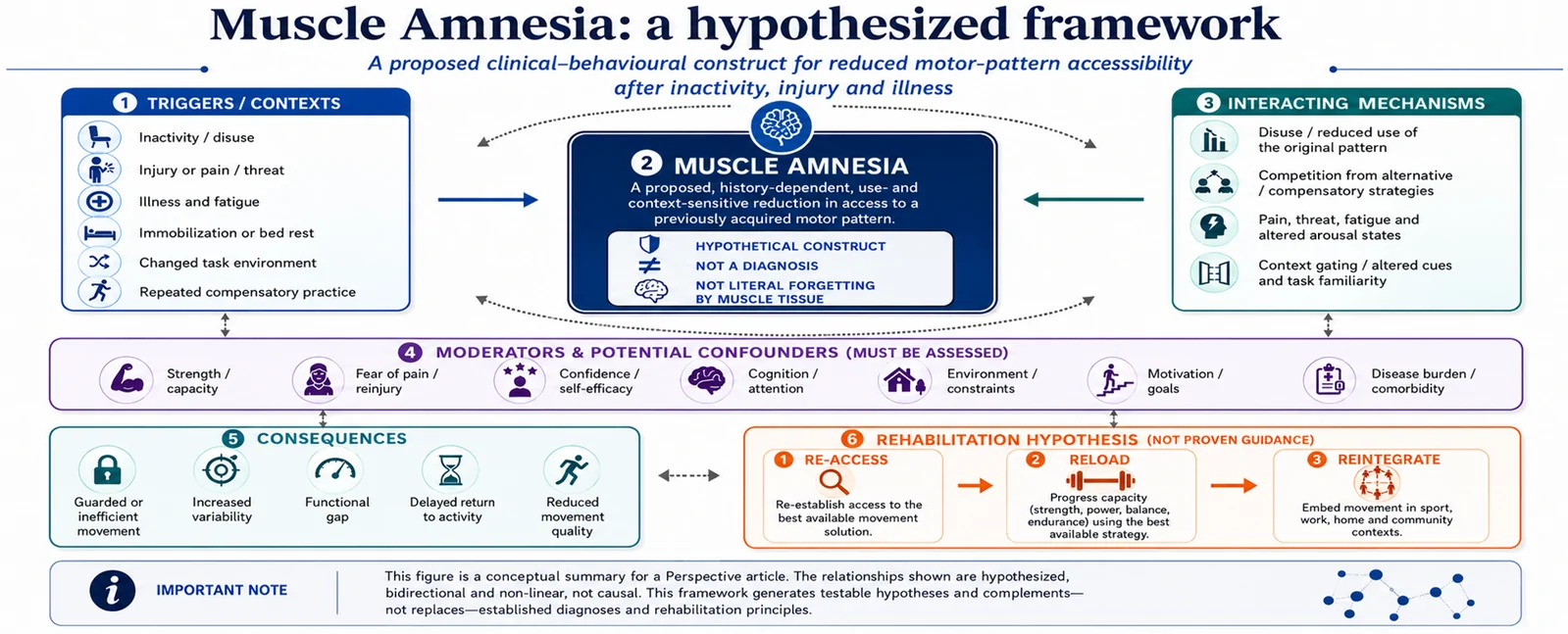
Muscle amnesia as a hypothesized framework for reduced motor-pattern accessibility after inactivity, injury and illness. The model is intentionally non-linear: altered exposure, compensatory strategies, motor-control mechanisms and biopsychosocial moderators may interact bidirectionally. The proposed rehabilitation sequence—screen, re-access, fade feedback, reload and reintegrate—is an intervention hypothesis, not a validated protocol.

Several terms require precision. A movement pattern refers to the observable organization of a task: sequencing, timing, joint coupling, stabilization, sensory prediction and muscle activation across segments, consistent with motor-learning and motor-control accounts that emphasize distributed neural control, internal models and task-specific coordination (8–11). A movement strategy or task solution is a broader term that includes the person's selected way of solving the task under current constraints; it may be efficient, protective, compensatory or simply available (5, 6). Motor-pattern accessibility refers to the probability that a previously acquired task solution can be selected and expressed when relevant cues, constraints and goals are present, a formulation consistent with evidence that practice, context, familiarity and competing learning shape motor expression (12–15). Movement quality is used here as an umbrella term for task-relevant features such as coordination, smoothness, stability, symmetry, timing, precision, efficiency and absence of maladaptive compensation; these dimensions should be measured explicitly because variability can be adaptive, asymmetry can be functional and compensation can be appropriate during healing (16–18). Therefore, movement quality should be interpreted against the task goal, the individual's capacity and the clinical context.

History-dependence means that the claim requires evidence, or at least credible history, that the person previously performed the target task more effectively. The relevant history may include both the individual learning history and the degree of automation, because motor skills are acquired, consolidated and retained across different time scales and degrees of familiarity (8, 15, 19, 20). A highly automated task may persist differently from a recently learned task, while a task learned only superficially may fail because it was never robustly acquired. Use-dependence refers to changes in task exposure: non-use, low-dose use, altered loading or repeated practice of a different strategy, all of which may interact with disuse-related neuromuscular change and learning competition (12, 21). Context-sensitivity refers to the fact that the same task may be expressed differently across sensory, emotional, environmental or social conditions; for example, a person may access a movement strategy in the clinic but not at home, or during slow practice but not under speed, fatigue or threat (13, 14, 22).

The intended scope is exercise rehabilitation after inactivity, non-progressive injury, reversible or partially reversible illness, hospital-associated deconditioning, pain-limited activity and return to sport or daily function (7, 23, 24). The construct should be applied cautiously in progressive neurological disease, severe cognitive impairment, advanced cachexia, uncontrolled pain, major structural limitation or severe cardiopulmonary restriction, where altered movement may be better explained by disease burden, capacity loss, compensatory recovery or safety constraints (4, 5).

## The term “Muscle Amnesia”

The term Muscle Amnesia is imperfect, and the risk of misunderstanding is real. “Muscle” can imply peripheral tissue, while the framework concerns the integrated neuromuscular system. “Amnesia” can imply literal forgetting, while the proposed problem is reduced access, selection or expression. We retain the term for three reasons. First, it is already clinically intelligible: patients and athletes often describe the problem as the body not remembering how to move. Second, it provides a counterpart to the widely used, equally imperfect term muscle memory, which can refer to cellular, epigenetic and neural persistence rather than memory within muscle alone (25, 26). Third, it functions as a practical label for a testable rehabilitation question: is the person unable to produce the movement because capacity is insufficient, or because a previously acquired task solution is currently difficult to access?

To avoid overclaiming, the scientific term beneath the label should remain reduced motor-pattern accessibility. Muscle Amnesia should be reserved for cases in which prior competence, altered-use history, disproportionate coordinative impairment and cue-responsive re-expression are all plausible. It should not be used as a synonym for weakness, poor technique, fear, deconditioning or low motivation.

## Relationship to existing constructs

Muscle Amnesia overlaps with, but does not replace, several established concepts. Compared with detraining, it focuses less on loss of capacity and more on whether capacity can be expressed through an appropriate task solution. Compared with learned non-use, it is not restricted to neurological impairment and does not require paresis; it may occur after orthopaedic injury, immobilization, pain, illness or prolonged inactivity. Compared with pain-adapted movement, it includes pain and threat as possible mechanisms but also includes disuse, fatigue, context mismatch and competing practice. Compared with skill decay, it emphasizes partial accessibility and cue responsiveness rather than simple loss of performance. Compared with maladaptive compensation, it asks whether a previously acquired alternative remains available and whether the current strategy dominates because of altered exposure history.

The unique contribution is therefore not mechanistic exclusivity. The construct is useful only if it integrates these mechanisms around one applied problem: the mismatch between recovering capacity and reduced access to task-relevant movement. This distinction has implications for assessment and treatment. If the primary problem is capacity, progressive loading may be sufficient. If the problem includes reduced motor-pattern accessibility, rehabilitation may need a brief reactivation phase before, or alongside, loading.

## Motor-control rationale: internal models, context and biopsychosocial constraints

A motor-control perspective strengthens the framework. Internal-model theories propose that the nervous system predicts the sensory consequences of action and uses those predictions for planning, control and learning (10, 11). These models are continually updated through practice, error, sensory feedback and task constraints. If an injury, illness or period of inactivity changes available sensory information, perceived threat or movement goals, the motor system may update toward a safer or less costly solution. Such updating is adaptive in the short term but may reduce accessibility of the prior strategy.

Motor memory research also suggests that practice, consolidation, task familiarity and context influence whether a learned solution is expressed. Motor skills are supported by distributed cortical, striatal, cerebellar and hippocampal-linked networks (8, 9, 15, 27). Long-term motor memory may combine action and context, preserving learned behaviour while making expression conditional on task conditions (14, 22). Therefore, a previous movement strategy may be retained but not preferentially selected when the body, environment or perceived risk has changed.

This interpretation remains hypothetical. A rapid response to cueing does not prove retrieval of an old motor representation. It may reflect attention, motivation, practice effects, reduced fear, task familiarization or adoption of a new strategy. For that reason, cue responsiveness should be treated as a screening signal rather than mechanistic evidence. Demonstrating re-expression would require pre-injury or pre-illness recordings, longitudinal data, kinematics, electromyography, intersegmental coordination, variability analysis and retention after cue withdrawal.

A biopsychosocial view is also necessary. Pain, fatigue, confidence, expectations, fear of reinjury, kinesiophobia, attentional focus, self-efficacy, cognitive load and the therapeutic environment can shape movement selection. Psychological readiness is already recognized in return-to-sport decisions (7, 24). Muscle Amnesia should therefore not be explained solely as a motor-memory problem. It is better understood as a candidate profile emerging from interactions between physical capacity, motor-control processes, perception, context and behaviour.

## Assessment: from clinical suspicion to research classification

The framework should separate three levels of use. First, clinical suspicion occurs when a therapist or exercise professional notices a disproportionate gap between capacity and task execution. Second, research classification requires pre-specified candidate criteria, confounder assessment and reproducible measurement. Third, outcome evaluation tests whether an intervention changes movement quality, retention, transfer, function or patient confidence. Conflating these levels risks circular reasoning (Table 1).

**Table 1:** Candidate criteria for a muscle amnesia profile and safeguards against overdiagnosis.

Domain	Clinical screening question	Research classification indicator	Confounders/exclusions	Expected supportive finding
Prior competence	Was the task previously performed better?	Video, baseline testing, competition/training record, therapist report, or credible patient history.	No prior learning; task was never automated; previous strategy was unsafe or not relevant.	The person has a plausible previously acquired task solution to compare against.
Altered-use history	Was there a period of non-use, altered loading, pain, illness, immobilization or compensatory practice?	Defined exposure window; documented training interruption, hospitalization, injury, pain episode or modified practice.	Progressive neurological disease, severe systemic disease burden, or structural limitations that fully explain movement change.	A plausible period existed in which a competing or protective solution could dominate.
Disproportionate coordinative impairment	Is movement organization worse than expected from strength, range and pain alone?	Task-specific kinematics, EMG timing, variability, smoothness, stability, step or joint coordination, video score.	Severe weakness, uncontrolled pain, contracture, fatigue, cognitive impairment, vestibular or cardiopulmonary limitation.	Movement-quality loss exceeds capacity loss, after relevant confounders are assessed.
Cue responsiveness and retention	Does performance improve with task-specific cues, and does some improvement remain after cue withdrawal?	Standardized re-exposure protocol; repeated trials; feedback withdrawal; transfer to related task; short-term retention testing.	Practice effect, motivation, attention, reduced fear, trial-to-trial variability, or adoption of a completely new strategy.	Improvement persists beyond immediate cueing and/or transfers to a relevant functional context.
Clinical relevance	Does the altered strategy limit function, progression, confidence or safety?	Patient-reported confidence, task performance, return-to-activity outcome, falls/near-miss history, progression tolerance.	Natural variation, adaptive compensation during healing, or harmless individual style.	Targeting movement accessibility changes rehabilitation decisions or outcomes.

Movement-quality assessment should be operational rather than impressionistic. Depending on the task, relevant indicators may include joint angles, timing, segment coordination, variability across repeated trials, smoothness, step width, step-time variability, trunk displacement, landing mechanics, force distribution, co-contraction, electromyographic onset timing, or energy cost. Multi-sensor and wearable approaches can support the measurement of movement quality, although definitions and processing methods vary (17). Inertial measurement units are increasingly used in rehabilitation decision-making and reporting recommendations are emerging for joint kinematics based on wearable sensors (17, 18). Video analysis remains useful when technology is limited, but ratings should be task-specific and anchored to observable criteria.

Cue responsiveness should be tested with caution. Immediate improvement after cues or feedback is not sufficient. More informative evidence would include improvement that is retained after feedback withdrawal, transfers to a related task, persists across sessions and resembles the individual's previous movement if such data exist. Equally important is identifying non-MA explanations: pain reduction, fear reduction, fatigue recovery, increased attention or natural trial-to-trial variability. Table 1 summarizes candidate screening and research criteria.

## Rehabilitation implications: an intervention hypothesis

We propose re-access, reload and reintegrate as an intervention hypothesis, not as a validated protocol (Figure 1). Many contemporary rehabilitation programmes already combine task-specific practice, feedback, progressive strengthening, graded exposure and functional reintegration. The added contribution of the Muscle Amnesia framework is sequencing and emphasis: when reduced motor-pattern accessibility is suspected, the clinician first tests whether a previously successful task solution can be re-expressed under low load before escalating physical demand.

The re-access phase is brief, low-load and high-feedback. It may include 2-5 sets of 3-8 high-quality repetitions of the target task or a simplified version, selected according to fatigue, pain and disease stage. The clinician may use external focus cues, foot-placement targets, tempo cues, tactile feedback, mirror or video feedback, rhythmic auditory cues, environmental constraint modification, or task simplification. Feedback should be concise and gradually withdrawn to test retention. Progression to reloading should require acceptable pain or threat, stable task execution across repeated trials, reduced need for cueing, and no major deterioration under mild fatigue or context variation. Evidence from motor-learning research supports the importance of feedback structure and attentional focus, although direct evidence for a Muscle Amnesia protocol is absent (28).

The reload phase progressively challenges the re-accessed task solution through strength, power, balance, endurance or sport-specific loading. The reintegrate phase embeds the task into meaningful contexts: home, work, stairs, uneven ground, training, competition or community mobility. In older adults and medical patients, this approach should coexist with strategies that address hospital-associated functional decline and deconditioning (23, 29). In sport, it should complement criterion-based return-to-sport frameworks that already consider strength, movement quality, psychological readiness and sport demands (7).

## Research agenda

The construct requires both exploratory and confirmatory research. Qualitative studies should come first in some populations. Interviews with athletes after injury, older adults after hospitalization and patients after cancer or chronic illness could examine whether people experience a perceived loss of familiar movement, what language they use, which tasks are affected, and whether the experience relates to fear, fatigue, pain or confidence. These studies would prevent the framework from being imposed only from the clinician's perspective.

Experimental studies could then test mechanisms. Healthy young or older adults could learn a standardized task such as sit-to-stand, stepping, squat, reaching or gait initiation to criterion. After acquisition, groups could undergo pure non-practice, non-practice plus constrained compensatory practice, or altered-context practice. Primary outcomes would include initial kinematic error, intersegmental coordination, variability, EMG timing, retention after cue withdrawal and relearning slope over 1-2 weeks. The Muscle Amnesia hypothesis predicts greater coordinative disruption and slower return of the original task solution after compensatory practice than after pure non-practice, despite similar capacity changes.

Clinical trials should be pragmatic but controlled. Populations could include patients after lower-limb injury, hospitalized older adults with functional decline, or people returning to activity after cancer treatment or chronic illness. Inclusion criteria should require credible prior competence, altered-use history, capacity assessment and reproducible movement-quality impairment. A trial could compare usual exercise rehabilitation with the same programme preceded by a standardized 1-2 week motor-reactivation module. Assessors should be blinded where feasible. Primary outcomes could include task-specific movement quality, retention after cue withdrawal, transfer to untrained tasks and patient-reported confidence. Follow-up should test whether early improvements persist at 4-12 weeks. Negative findings would be valuable; if capacity-only training performs equally well, or if candidate cases cannot be distinguished reproducibly, the construct should be narrowed or abandoned.

## Limitations and safeguards

This framework has important limitations. First, it overlaps with existing constructs including detraining, learned non-use, skill decay, contextual interference, pain adaptation, fear avoidance and compensation. Its value depends on whether the motor-pattern accessibility lens improves explanation or rehabilitation decisions beyond those terms. Second, there is no biomarker and no validated diagnostic test. Third, original movement patterns are often unknown, unavailable or not necessarily ideal. A previously successful movement strategy may have been effective for the task but not optimal or safe under new constraints.

Fourth, cue responsiveness has limited specificity. Improvement after instruction may reflect attention, practice, motivation, reduced fear or adoption of a new strategy rather than re-access of a previous one. Fifth, the terminology could medicalize normal movement variation or encourage unnecessary correction of adaptive compensations. Clinicians should not try to make every person move according to a single ideal template. The relevant question is whether the current strategy limits function, safety, confidence or progression relative to the person's goals and capacity. Finally, in serious illness or advanced cachexia, movement changes may be dominated by systemic disease, fatigue, malnutrition, inflammation, neuropathy or cardiopulmonary limitation. In such cases, movement reactivation can only be one small component of multimodal care.

## Conclusion

Muscle Amnesia is proposed as a cautious and testable term for reduced motor-pattern accessibility after inactivity, injury or illness. It does not imply literal forgetting by muscle tissue, and it should not be treated as a diagnosis. Its purpose is to draw attention to a practical rehabilitation question: when capacity returns, can the person access a task-relevant movement strategy that gives that capacity functional meaning? The revised framework predicts a candidate profile characterized by prior competence, altered-use history, disproportionate coordinative impairment, context sensitivity and cue responsiveness that persists after feedback withdrawal. Whether this profile exists as a useful construct remains to be tested. If confirmed, rehabilitation may benefit from explicitly re-accessing movement before escalating load. If refuted, the concept should be narrowed or discarded.

## Data Availability

The original contributions presented in the study are included in the article; further inquiries can be directed to the corresponding author.
